# Fatty acids are crucial to fuel NK cells upon acute retrovirus infection

**DOI:** 10.3389/fimmu.2023.1296355

**Published:** 2023-11-29

**Authors:** Simone Schimmer, Daniela Mittermüller, Tanja Werner, Paul E. Görs, Sven W. Meckelmann, David K. Finlay, Ulf Dittmer, Elisabeth Littwitz-Salomon

**Affiliations:** ^1^ Institute for Virology, University Hospital Essen, University of Duisburg-Essen, Essen, Germany; ^2^ Institute for Experimental Immunology and Imaging, University Hospital Essen, University of Duisburg Essen, Essen, Germany; ^3^ Applied Analytical Chemistry, University of Duisburg‐Essen, Essen, Germany; ^4^ School of Biochemistry and Immunology, School of Pharmacy and Pharmaceutical Sciences, Trinity Biomedical Sciences Institute, Trinity College Dublin, Dublin, Ireland; ^5^ Institute for Translational HIV Research, University Hospital Essen, University of Duisburg-Essen, Essen, Germany

**Keywords:** NK cells, lipid metabolism, retrovirus, beta-oxidation, migration, fatty acids, cytotoxicity, CD36

## Abstract

Natural killer (NK) cells are cytotoxic innate immune cells, able to recognize and eliminate virus-infected as well as cancer cells. Metabolic reprogramming is crucial for their activity as they have enhanced energy and nutritional demands for their functions during an infection. Fatty acids (FAs) represent an important source of cellular energy and are essential for proliferation of immune cells. However, the precise role of FAs for NK cells activity in retrovirus infection was unknown. Here we show that activated NK cells increase the expression of the FA uptake receptor CD36 and subsequently the uptake of FAs upon acute virus infection. We found an enhanced flexibility of NK cells to utilize FAs as source of energy compare to naïve NK cells. NK cells that were able to generate energy from FAs showed an augmented target cell killing and increased expression of cytotoxic parameters. However, NK cells that were unable to generate energy from FAs exhibited a severely decreased migratory capacity. Our results demonstrate that NK cells require FAs in order to fight acute virus infection. Susceptibility to severe virus infections as it is shown for people with malnutrition may be augmented by defects in the FA processing machinery, which might be a target to therapeutically boost NK cell functions in the future.

## 1 Introduction

Natural killer (NK) cells are cytotoxic immune cells that eliminate virus-infected as well as cancerous cells. As cells of the innate immune system, NK cells recognize target cells with their germline-encoded receptors and do not require priming. Activated NK cells release cytotoxic granules into immunological synapses that contain the pore-forming protein perforin and cytolytic granzymes (Gzm), which facilitate rapid induction of cell death in target cells (1). The expression of death receptor ligands such as Tumor necrosis factor (TNF)-related apoptosis-inducing ligand (TRAIL) or Fas ligand (FasL) is another mechanism of NK cells to eliminate target cells (2). Additionally, NK cells produce cytokines such as interferon (IFN)-γ and TNF that mediate direct antiviral functions or modulate the function of innate and adaptive immune cells (3). Similar to other immune cells, NK cells circulate through multiple tissues ready to eliminate target cells, thus facing various conditions of nutrients and oxygen availability. This requires the ability to reprogram the cellular metabolism within the extracellular environment to provide sufficient energy as well as biosynthetic precursors for their diverse functions (4 ). Immune cells need the energy-carrying molecule adenosine triphosphate (ATP) for most cellular processes. ATP is produced through the processes of glycolysis and/or oxidative phosphorylation (OXPHOS).(5). In the process called glycolysis glucose becomes converted into pyruvate through multiple enzymatic steps in the cytosol and generates two molecules of ATP. There are two alternate fates for pyruvate. First, it can be converted into lactate and secreted, or second, can be converted to acetyl-CoA, which is metabolized via metabolic cycles such as the TCA cycle or the Citrate Malate Shuttle (6, 7) in the mitochondria to generate two key reducing agents, reduced nicotinamide adenine dinucleotide (NADH) and flavin adenine dinucleotide (FADH2), which feed electrons into the electron transport chain and to its end the ATP synthase converts adenosine diphosphate (ADP) to ATP. This generates a net of approximately 36 molecules of ATP per molecule of glucose. Despite the macronutrient glucose, amino acids as well as FAs can be used to fuel lymphocyte functional demands, which is of special importance in nutrient-deprived tumor environment (8). FAs can be used to generate ATP in a process called β-oxidation or for lipid synthesis to build new membranes. Key enzyme for the transport of FAs into the mitochondria is carnitine palmitoyltransferase 1 (CPT-1), which can be inhibited by careful administration of low doses of etomoxir (4, 9, 10). β-oxidation occurs in the mitochondria and is a multistep process in which FAs are broken down. Acetyl-CoA generated by the β-oxidation is used to generate ATP in mitochondrial TCA cycle (9). Free FAs are energy-dense molecules as the complete oxidation of palmitate (16 carbons) results in a minimum net of 106 ATP molecules (11). The preferential utilization of β-oxidation is connected to specific T cell fates and functional specifications. It has been shown that β-oxidation is essential for generation of memory CD8^+^ T cells (12). Interestingly, the generation of the suppressive population of CD4^+^ T cells (Tregs) require lipid oxidation whereas effector CD4^+^ T cells are suppressed by FA oxidation (13). Activation of AMP-activated kinase (AMPK), a metabolic regulator and the antagonist of mechanistic target of rapamycin (mTOR), promotes enhanced FA oxidation and subsequently an enrichment of Tregs (13). Tregs take up externally derived FAs contradictory to effector CD4^+^ T cells or memory CD8^+^ T cells (14, 15). These studies show the importance of lipid metabolism for T cells but whether NK cells utilize FAs to fuels their demands is not completely understood. A previous *in vitro* study showed that treatment of NK cells with high dose etomoxir (300 μM), which prohibits β-oxidation, did not change the ATP concentration after cytokine or receptor stimulation in NK cells (16). Interestingly, a long-term excess of FAs as seen in obesity or FA-rich lymphoma had detrimental effects on IFN-γ-producing NK cells and their cytotoxicity (17, 18). Here we used the Friend retrovirus (FV) to infect mice and determined the lipid metabolism in NK cells during acute infection. FV consist of two parts; of the replication-competent but apathogenic F-MuLV (Friend Murine Leukemia Virus) and the replication-defective but pathogenic SFFV (spleen focus-forming virus) (19). Adult C57BL/6 mice infected with FV develop a strong immune response and control viral spread, but were unable to clear infection leading to chronic infection. We have shown before that NK cells are important to restrict viral replication during acute FV infection analog to NK cells in HIV or SIV infection (20). Ablation of NK cells in acute FV infection resulted in increased viral loads (21). Similar to NK cells in HIV infection, NK cells become dysfunctional in chronic FV infection (21, 22), but as seen in SIV infection a population of adaptive, antigen-specific NK cells arise in FV infection (23, 24). Recently we have shown that NK cells reprogram several metabolic parameters such as their mitochondrial polarization and mass, glycolysis as well as OXPHOS during acute retrovirus infection (25). However, nothing is known about the use of free FAs for NK cells antiviral functions, which might be used as therapeutic approach to boost NK cell responses in the future. Thus, FV model is a suitable experimental model to determine the NK cells FA metabolism during acute retrovirus infection. Here, we demonstrate that NK cells increase the uptake of free FAs and NK cells expressing the fatty acid uptake receptor CD36 exhibited strong proliferation, cytotoxicity, cytokine production as well as metabolic changes. In acute retroviral infection, NK cells show a strong capacity to break down FAs for ATP generation. Inhibition of β-oxidation resulted in severely compromised elimination of target cells by NK cells. Finally, the inhibition of FA breakdown had a negative influence on the migratory capacity of NK cells. These results demonstrate that metabolic rewiring of antiviral NK cells is important and may facilitate the development of innovative therapies that target the cellular metabolism of antiviral NK cells.

## 2 Materials and methods

### 2.1 Mice and FV infection

Experiments involved sex- and age-matched inbred C57BL/6 mice from Harlan Laboratories, Germany, maintained in a pathogen-free environment. The mice were a minimum of 7 weeks old at the start of the experiments. The FV complex, consisting of B-tropic Friend murine leukemia helper virus and polycythemia-inducing spleen focus-forming virus, was injected. The FV stock was prepared as a 15% spleen cell homogenate from susceptible BALB/c mice infected for 14 days with 4,000 SFFU of FV. Mice received intravenous injections of 0.1 ml phosphate-buffered saline (PBS) containing 40,000 SFFU of FV. Notably, the virus stock did not contain lactate dehydrogenase-elevating virus. All experiments were approved and conducted in compliance with the State Agency for Nature, Environment, and Consumer Protection (LANUV).

### 2.2 Flow cytometry stainings

Spleens were homogenized and washed with PBS. Splenocytes were plated onto 96 well plates and stained for 15 min at room temperature in the dark. Cells were washed and acquired at multi-parameter flow cytometer or fixed for further intracellular (Fixation/Permeabilization Solution Kit, BD Biosciences) or intranuclear stainings (Foxp3 Transcription Factor Fixation/Permeabilization kit, ThermoFisher). For cytokine stainings, splenocytes were restimulated with ionomycin (500 ng/ml), phorbol myristate acetate (PMA; 25 ng/ml), monensin (1X, BioLegend), and brefeldin A (2 μg/ml) diluted in RPMI buffer at 37°C for 3 h. Multi-parameter flow cytometry was done with the following antibodies: CD3 (clone 145-2C11, Thermo Fisher), CD36 (HM36, BioLegend), CD49b (DX5, Thermo Fisher), CD69 (H1.2F3, BioLegend), GzmB (GB11, Becton Dickinson), IFN-γ (XMG1.2, Thermo Fisher), NK1.1 (PK136, Becton Dickinson), pS6 (D57.2.2E, Cell Signaling). HCS LipidTOX Green (ThermoFisher) was stained 1:1000 after fixation of cells before acquisition.

### 2.3 Detection of cellular metabolism using SCENITH

RPMI1640 with 10% Fetal Bovine Serum and 100 U/ml Penicilin/100 µg/ml Streptomycin was prewarmed and splenocytes were added in duplicate on 96 well plates. Inhibitors were added (final concentrations: 2-DG 100 mM, oligomycin 1 µM, etomoxir 5 µM, 40 µM) and incubated for 30 min at 37°C. Puromycin was added (final concentration 10 µg/ml, Sigma) for 20 min. Cells were washed with cold PBS and FC block (BD Biosciences) was performed at 4°C for 5 min. Cell surface was stained at 4°C for 30 min in the dark and cells were washed. Cells were fixed with Fixation/Permeabilization Solution Kit (BD Biosciences). Anti-puromycin (Sigma, clone 12D10) staining was performed in perm-buffer at 4°C for 1 h. Cells were measured at BD Canto II. Glucose dependency and fatty acid capacity was calculated by using the geoMFI: (no inhibitor – 2-DG)/(no inhibitor – 2-DG Oligo). Mitochondrial dependency and glycolytic capacity was calculated by using the geoMFI: (no inhibitor – oligomycin)/(no inhibitor – 2-DG Oligo).

### 2.4 Fatty acid uptake

Splenocytes were plated on 96 well plates. Bodipy FL C16 (4,4-Difluoro-5,7-Dimethyl-4-Bora-3a,4a-Diaza-s-Indacen-3-Hexadecanoacid, Invitrogen) was diluted in supplemented RPMI1640 in 12,5 µM (final concentration) and added to wells. Incubation at 37°C for 30 min in a humidified atmosphere with 5% CO_2_. Cells were stained and measured at BD Canto II.

### 2.5 *In vitro* kill assay

NK cells were extracted from spleens using the MojoSort Mouse NK cell Isolation Kit from BioLegend, following the manufacturer’s instructions. YAC-1 target cells, labeled with carboxyfluorescein succinimidyl ester (CFSE, 2.5 μM), were co-incubated with NK cells at an E:T ratio of 25:1 in prewarmed 1xDMEM (Sigma D2429, supplemented with 3.7 g/L sodium bicarbonate, 10% FCS, 50 μM β-mercaptoethanol). The co-incubation occurred in 96-well U-bottom plates at 37°C in a humidified 5% CO2 atmosphere. After 4 hours, cells were washed and subsequently stained with a fixable viability dye. Fluorescence was acquired at BD Canto II. Killing was calculated with (dead tumor cells)/(all tumor cells) x 100.

### 2.6 Incubation of activated NK cells with etomoxir

Splenocytes were lysed for erythrocytes and plated in 96 well plates. Cells were cultured for two days in total with IL-15 (12,5 ng/ml) and when indicated, etomoxir (5 µM, 40 µM) were added to culture. IL-2 (20 ng/ml) and IL-12 (10 ng/ml) were added for the last 24 hours of IL-15 incubation. Cells were stained and acquired at flow cytometer.

### 2.7 Conjugate assay

NK cells were isolated using MojoSort Mouse NK cell Isolation Kit from BioLegend and stained with Tag-It Violet (BioLegend, 25 µM final). YAC-1 cells were labeled with CFSE from BioLegend (final concentration 5 µM) and used in an E:T ratio of 1:2. Etomoxir (40 µM) was added to co-culture. Cells were centrifuged for 1 min at 20 g and co-incubated for 1 min and 60 min at 37°C. After gentle mixing, ice-cold PFA (0.5%) was added and cells were measured at flow cytometer. Calculation was performed by dividing the conjugates (CFSE^+^Violet^+^) through all NK cells (Violet^+^), multiplied by 100.

### 2.8 Migration of NK cells

Bead isolated NK cells (MojoSort, BioLegend) were added in the upper compartment of transwells (pore size 3 µM, Costar). Enriched samples contained around 77% NK cells. YAC-1 cells were seeded in the lower compartment. Cells were seeded in an E:T ratio of 1:2 in 1xDMEM for 4 hours at 37°C. Etomoxir (40 µM) was added in both compartments, if indicated. Cells were stained and NK cells were analyzed and flow cytometer.

### 2.9 Real-time PCR

NK cells were isolated using magnetic beads from BioLegend and frozen in DNA/RNA Shield from Zymo Research. Enriched samples contained at least 77% NK cells. RNA was isolated with RNeasy Micro Kit from Qiagen. cDNA was synthesized with SCRIPT cDNA Synthesis Kit from Jena Biosciences. The real-time PCR was conducted in duplicate using the innuMIX qPCR DSGreen Standard (Innuscreen) and the Rotor-Gene Q (Qiagen). The quantitative mRNA levels were normalized to β-actin mRNA expression levels. The following oligonucleotide sequences were used: β-actin 5’-aaatcgtgcgtgacatcaaa-3’, 5’‐caagaaggaaggctggaaaa-3; PPARα 5’ CGGGAAAGACCAGCAACAAC 3’, 5’ TGGCAGCAGTGGAAGAATCG 3’; PPARγ 5′- GTACTGTCGGTTTCAGAAGTGCC -3′, 5′- ATCTCCGCCAACAGCTTCTCCT 3′; SCARB1 5′- ACACCCGAATCCTCGCTGGAAT -3′, 5′- CCGTTGGCAAACAGAGTATCGG -3′; SCARB2 5′- TAGCCAACACCTCCGAAAACGC -3′, 5′- CGAACTTCTCGTCGGCTTGGTA -3′; FABP5 5′-GACGACTGTGTTCTCTTGTAACC-3′, 5′- TGTTATCGTGCTCTCCTTCCCG-3′; CD36 5′-GGACATTGAGATTCTTTTCCTCTG-3′, 5′-GCAAAGGCATTGGCTGGAAGAAC -3′; CPT1α 5′- GGCATAAACGCAGAGCATTCCTG-3′, 5′-CAGTGTCCATCCTCTGAGTAGC-3′;

### 2.10 Gas chromatography-atmospheric pressure chemical ionization-mass spectrometry (GC-APCI-MS) analysis

Caprylic acid (FA 8:0), myristic acid (FA 14:0), palmitic acid (FA 16:0), palmitoleic acid (FA 16:1 Δ9), stearic acid (FA 18:0), oleic acid (FA 18:1 Δ9), linoleic acid (FA 18:2 Δ9,12), and linolenic acid (FA 18:3 Δ9,12,15) were purchased from Sigma-Aldrich (Traufkirchen, Germany). Decanoic acid (FA 10:0), lauric acid (FA 12:0), arachidic acid (FA 20:0), arachidonic acid (FA 20:4 Δ5,8,11,14), eicosapentaenoic acid (FA 20:5 Δ5,8,11,14,17), docosanoic acid (FA 22:0), docosapentaenoic acid (FA 22:5 Δ7,10,13,16,19), docosahexaenoic acid (FA 22:6 Δ4,7,10,13,16,19), lignoceric acid (FA 24:0), hexacosanoic (FA 26:0) acid as well as the stable isotope labled internal standards ^2^H_2_-decanoic acid (^2^H_2_-FA 10:0), ^2^H_2_-pentadecylic acid (^2^H_2_-FA 15:0), ^2^H_4_-stearic acid (^2^H_4_-FA 18:0), ^2^H_8_-arachidonic acid (^2^H_8_-FA 20:4 Δ5,8,11,14), and ^2^H_4_-lignoceric acid (^2^H_4_-FA 24:0) were purchased from Cayman Chemical Company (Hamburg, Germany). 2,3,4,5,6-Pentafluorobenzyl bromide (for GC derivatization; ≥98.5%) and N,N‐diisopropylethylamine (≥99.5%) were purchased from Sigma-Aldrich (Traufkirchen, Germany). Dichloromethane (≥99.8%) was purchased from Thermo Fisher Scientific (Schwerte, Germany), methyl tert-butyl ether (MTBE; LC-MS grade), and acetic acid (LC-MS grade) were purchased from Merck (Darmstadt, Germany), methanol (LC-MS grade) was purchased from Avantor (Darmstadt, Germany). Potassium hydroxide (pro analysi) was purchased from Bernd Kraft GmbH (Duisburg, Germany). Ultrapure water with a resistivity of 18.2 M Ω/cm was desalted and filtered by a Sartorius Stedim water purification system (Sartorius, Goettingen, Germany). For lipid extraction, a 5 µL serum sample was combined with a 100 µL methanol solution containing internal standards, including ^2^H_2_-decanoic acid (^2^H_2_-FA 10:0), ^2^H_2_-pentadecylic acid (^2^H_2_-FA 15:0), ^2^H_4_-stearic acid (^2^H_4_-FA 18:0), ^2^H_8_-arachidonic acid (^2^H_8_-FA 20:4 Δ5,8,11,14), and ^2^H_4_-lignoceric acid (^2^H_4_-FA 24:0), each at a concentration of 2,000 nM. To hydrolysis complex lipids, 60 µL of 10 M potassium hydroxide was added to the mixture. The samples were homogenized in an ultrasonic bath for 5 minutes at 0°C and then incubated at 60°C for 30 minutes to ensure complete hydrolysis. The solution was neutralized by adding 70 µL of 50% acetic acid (v/v) while keeping the samples on ice. Afterwards, the samples were subjected to lipid extraction based on the method outlined by Matyash et al. (26), with some modifications. This involved the addition of 300 µL of methanol, followed by vortexing for 1.5 minutes. Subsequently, 600 µL of methyl tert-butyl ether (MTBE) was added and the mixture was vortexed for another 1.5 minutes. Phase separation was induced by the addition of 300 µL, and the mixture was again vortexed for 1.5 minutes. The upper phase, containing the fatty acids, was then collected. The aqueous phase was washed with 300 µL of MTBE and combined with the previously collected MTBE phases. Finally, the samples were dried using a vacuum evaporator at 30°C. The dried lipid extracts were reconstituted in 20 µL of a solution of 10% diisopropylethylamine in dichloromethane (1/9; v/v) with the addition of 10% 2,3,4,5,6 pentafluorobenzylbromide in dichloromethane (1/9; v/v). After the incubation at 50°C for 1 hour, the solvent was evaporated under vacuum at 30°C. The derivatized fatty acids were dissolved in 100 µL of methanol for subsequent analysis. Fatty acid analysis was conducted following the procedure described by Görs et al. (27). An Agilent 7890B GC, equipped with a liquid autoinjector and a DB-5 column (30 m x 250 μm x 0.25 μm) (Agilent Technologies, Waldbronn, Germany), was used for gas chromatographic analysis. A 1 µL sample was injected with a split ratio of 1:10 and a septum purge flow of 1.5 mL/min at 320°C. Helium was used as the carrier gas at a flow rate of 1.3 mL/min. The temperature gradient was as follows: initial 100°C, increased at 15°C/min to 160°C, followed by a linear increase of 5°C/min to 320°C, which was held for 5 minutes. The GC was coupled with an APCI source to a 6495 triple Quad mass spectrometer (Agilent Technologies, Waldbronn, Germany). The APCI used a corona current of 35 µA, a source gas flow of 11 L/min, a source gas temperature of 270°C, a nitrogen auxiliary gas flow of 1 L/min, and a capillary voltage of 2000 V. Negative ionization was used, and ions were monitored in pseudo SRM mode. Therefore, the m/z ratios of the [M-PFB]- ions of the corresponding fatty acid was set as m/z for Q1 and Q3, with no fragmentation energy applied. The cycle time was 300 ms.

### 2.11 Statistical analyses

GraphPad Prism version 8 was employed for graph preparation and statistical analysis. Statistical differences between two groups (naïve, 7 dpi) were assessed using either the Mann–Whitney test (nonparametric) or unpaired t-test (parametric). The analysis of multiple groups (naïve, FV, treated) utilized the nonparametric Kruskal–Wallis one-way analysis of variance (ANOVA) on ranks and Dunn’s multiple-comparison test (nonparametric), or ordinary one-way ANOVA with Tukey multiple-comparison test (parametric). Correlating variables, linear regression and Goodness of fit were determined.

## 3 Results

### 3.1 Activated phenotype and altered mTOR signaling in NK cells after acute retrovirus infection

**Figure 1:**
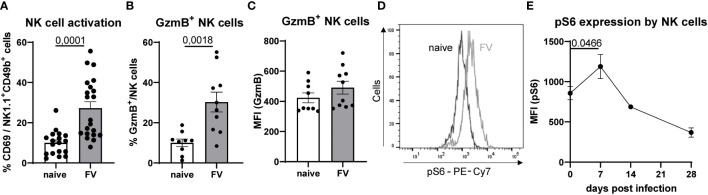
NK cell phenotype and pS6 expression upon retrovirus infection. Splenocytes of naïve and FV-infected animals (7 dpi) were stained for NK cell markers (NK1.1^+^, CD49b^+^, CD3^-^) and analyzed for activation (CD69, **A**) and cytotoxicity (GzmB, **B, C**). At least nine mice per group from three independent experiments were used. Statistically significant differences were analyzed by Mann-Whitney test **(A)** or unpaired t test **(B)**. NK cells were stained for pS6 in naïve and mice from 7 dpi, which expression is shown as a representative histogram in **(D)** A kinetic of NK cells expressing pS6 is shown in **(E)** NK cells were analyzed at 0 (22 mice), 7 (17 mice), 14 (9 mice) and 28 dpi (6 mice). NK cells from 0 and 7 dpi were compared and statistically significant differences were analyzed with an unpaired t test. Data are presented as mean values ± SEM.

C57BL/6 mice were infected with 40,000 Spleen Focus-Forming Units (SFFU) of FV and examined at 7 dpi. Splenic NK cells from both naïve and FV-infected mice were analyzed for activation by measuring CD69. As shown in **Figure 1A**, NK cell activation is approximately 2.7 times higher in FV-infected compared to naïve animals. In addition to the augmented activation of NK cells during acute infection, the percent of GzmB expressing NK cells was higher upon FV infection (**Figure 1B**). Nevertheless, the expression of GzmB on NK cells was not changed (**Figure 1C**). Increased cytotoxic activity of NK cells requires metabolic reprogramming. Thus, we determined the mTOR activity in NK cells, which is a master regulator that senses nutritional and environmental signals and promotes cellular growth. Here we analyzed phospho-S6 (pS6), a marker for the mTOR signaling pathway, over time in FV infection (**Figures 1D, 1E**). We found the peak of pS6 expression at 7 dpi, which was already reduced at 14 dpi (**Figure 1E**). These findings demonstrate that NK cells experience increased activation and undergo metabolic reprogramming during the acute phase of FV infection.

### 3.2 Increased NK cell capacity to metabolize FAs upon acute FV infection

**Figure 2:**
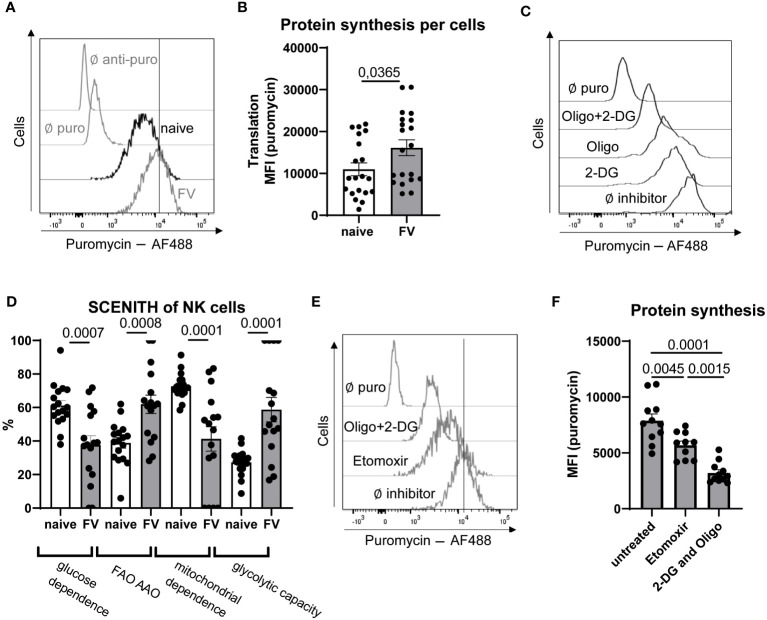
Analysis of NK cells ability to use FA as fuel after FV infection. C57BL/6 mice were infected with FV or used as naïve animals. A representative histogram of NK cells from naïve and FV-infected animals treated with puromycin (10 µg/ml) and stained with anti-puromycin antibody is shown in **(A)** The MFI of puromycin in NK cells was analyzed and is shown in **(B)** A minimum of 20 mice per group from seven independent experiments were used and statistically significant differences were analyzed by Mann-Whitney test. Splenocytes from naïve and FV-infected mice (7 dpi) were treated with oligomycin (1 µM), 2-DG (100 mM) or oligomycin plus 2-DG. A representative histogram of NK cells from naïve mouse with these treatments is shown in **(C)** Analysis of glucose dependency and FA oxidation (FAO) and amino acid oxidation (AAO) was analyzed by using 2-DG-treated cells in uninfected and infected mice **(D)**. Mitochondrial dependence as well as glycolytic capacity was calculated by using oligomycin-treated cells. At least 17 mice per group from six independent experiments were used. **(E)** NK cells are shown in a representative histogram of etomoxir (5 µM, 40 µM) or oligomycin (1 µM) plus 2-DG (100 mM) treated splenocytes. In **(F)** the puromycin MFI of NK cells treated with etomoxir or 2-DG plus oligomycin is shown. A minimum of ten values from two independent experiments were used. Statistically significant differences were analyzed by Ordinary One-Way ANOVA. Data are presented as mean values ± SEM.

Activated immune cells have higher energy demands compared to quiescent cells. Energy is provided in form of ATP to cells. We have generated an approximation of ATP demand in NK cells by using the SCENITH method (28). This method works based on the premise that the most energy demanding process in the cell is protein synthesis. Puromycin is added to the cell and is then incorporated into newly synthesized proteins. Puromycin incorporation was significantly higher in NK cells during acute infection compared to NK cells from naïve mice demonstrating that NK cells responding to acute infection have increased rates of protein synthesis and an increased demand for ATP to support the protein translation (**Figures 2A, B**). If in a parallel sample puromycin plus a mitochondrial inhibitor or glycolysis inhibitor is added, it will end in less puromycin incorporation because ATP is now limited due to the inhibition of mitochondrial activity or glycolysis. In this way you can gauge how a cell is making its ATP, whether it is through glycolysis, glucose-fueled mitochondrial OXPHOS or metabolism of FAs. To interrogate how the NK cells were generating their ATP, we first treated splenocytes with inhibitors to inhibit glycolysis (2-deoxyglucose, 2-DG) or inhibition of glycolysis and mitochondrial OXPHOS (oligomycin) together (2-DG+oligomycin), which provides a negative control of cells with extremely limited capacity to make ATP (**Figures 2C, D**). There was a strong reduction in puromycin incorporation in cells provided the inhibitor of glycolysis 2-DG indicating reduced ATP pools (**Figure 2C**). In the presence of 2-DG, the ability of the cell to make ATP through glycolysis or glucose-fueled mitochondrial OXPHOS is prevented. Under these conditions the remaining ATP is most likely generated through the mitochondrial oxidation of amino acids (AAO) or fatty acids (FAO). When cells are provided with puromycin plus both 2-DG and oligomycin, the processes of AAO and FAO are also blocked. Therefore, a substantial difference in the puromycin incorporation between the 2-DG condition and the 2-DG plus oligomycin condition indicates that the cells are engaging in significant levels of either AAO or FAO (**Figure 2D**). The fuel utilization in NK cells from naïve mice and those with acute FV infection was interrogated. Interestingly, glucose dependent ATP production (2-DG sensitive puromycin incorporation) was reduced in NK cells from acute infection with a concomitant increase in β-oxidation and/or amino acid oxidation in NK cells from infected animals, demonstrating the ability of NK cells to switch from glycolysis to FAs or amino acids as fuels (**Figure 2D**). Second, treatment of cells with oligomycin, which blocks the ATP production through mitochondrial OXPHOS, quantifies how much translation depends on OXPHOS. Then the difference between inhibiting OXPHOS alone and inhibition of OXPHOS plus glucose-fueled glycolysis (2-DG plus oligomycin) provides information about ATP-dependent protein synthesis that can be maintained in the absence of any mitochondrial generated ATP. This reflects the glycolytic capacity because cells increase glycolysis to the maximum possible rate in the absence of OXPHOS. Taken together, these data reveal two interesting insights. Firstly, NK cells from mice during acute infection have a significant increase in the glycolytic machinery allowing for a higher maximal glycolytic rate when required, such as if OXPHOS were inhibited *in vivo* due to hypoxia. Secondly, under conditions when mitochondrial OXPHOS is possible, NK cells show a switch in fuel preference away from glucose towards FA and/or amino acids during acute FV infection (**Figure 2D**).

Therefore, we next considered whether FAs are important fuels for NK cells during acute retroviral infection. Short- and medium-chain fatty acids with a chain length less than 12 carbons can freely diffuse into the mitochondrial matrix. However, the transport and subsequent oxidation of long-chain fatty acids with a chain length between 14 to 18 carbons are regulated by the carnitine palmitoyltransferase 1 (CPT-1) system (29). We treated splenocytes with low doses of etomoxir (5 μM, 40 μM), which is an irreversible inhibitor of CPT-1, and inhibited the transport of long-chain fatty acids into mitochondria. In previous studies it was demonstrated that high concentrations of etomoxir (up to 200 μM) had an influence on the proliferation of cells independent of β-oxidation (10). We have shown that the proliferation of NK cells was not altered by treating splenocytes with 5 µM or 40 µM etomoxir (**Supplementary Figure 2**). When low-dose of etomoxir was used to inhibit CPT-1, a decrease in puromycin incorporation was observed, though not to the level seen with 2-DG plus oligomycin. These data argue that inhibition of β-oxidation diminishes ATP levels in the cells and that FAs are an important fuel, along with glucose, used by NK cells to make ATP during acute retroviral infection (**Figures 2E, F**). Interestingly, etomoxir treatment significantly decreased the puromycin expression of NK cells from FV-infected animals almost to similar levels as 2-DG or oligomycin single treatment (**Supplementary Figure 1A**). This shows that FAs are major contributors to energy generation upon acute retroviral infection. NK cells from uninfected animals only slightly decreased the puromycin expression after etomoxir treatment. Moreover, NK cells from naïve animals strongly decreased the puromycin expression after single 2-DG or oligomycin treatment, demonstrating their strong dependence on glycolysis and OXPHOS independent of β-oxidation. To proof the influence of etomoxir on the NK cells energy generation, we analyzed the influence of low-dose etomoxir on the oxygen consumption rate (OCR) (**Supplementary Figure 1B**). Similar to SCENITH data, we did not observe a reduction of OCR after low-dose etomoxir treatment in NK cells from naïve animals, but observed a significant decrease of OCR after etomoxir treatment in NK cells isolated from FV-infected animals. Taken together, these data demonstrate that NK cells increase their ability to utilize FA as source of energy during acute infection.

### 3.3 Enhanced expression of FA uptake receptor CD36 and augmented uptake of FAs after acute FV infection

**Figure 3:**
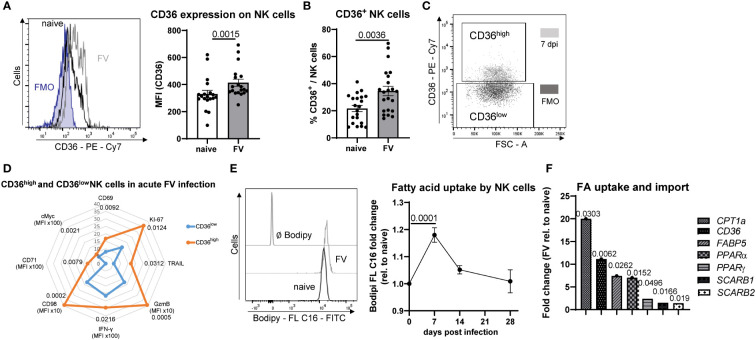
Uptake of fatty acids by NK cells upon acute retrovirus infection. C57BL/6 mice were infected with FV or used as uninfected controls. Spleens were harvested and homogenized. Splenocytes were stained and analyzed for CD36^+^ NK cells. A representative histogram of CD36^+^ NK cells as well as the expression of CD36 is shown in **(A)**. The percentage of CD36^+^ NK cells is shown in **(B)**. At least 19 mice from seven individual experiments were used. Statistically significant differences were analyzed by Mann-Whitney test **(A)** or an unpaired t test **(B)**. A representative graph of CD36 expressing NK cells and gates for CD36^high^ and CD36^low^ are shown in **(C)** CD36^high^ and CD36^low^ NK cells from FV-infected animals were analyzed for activation (CD69) and proliferation (KI-67), IFN-γ, cytotoxicity (GzmB, TRAIL) and several metabolic parameters (cMyc, CD71, CD98) and displayed at **(D)**. In **(E)** a representative histogram of NK cells positive for Bodipy FL C16 is shown. The uptake of FAs was analyzed in NK cells of naïve or FV-infected (7, 14, 28 dpi) animals by measuring Bodipy FL C16. At least ten mice were used in naïve group, 13 mice at 7 dpi, three mice at 14 dpi and four mice at 28 dpi. Statistically significant differences between naïve and 7 dpi were analyzed by an unpaired t test. Spleen NK cells were isolated from naïve or 7 dpi mice. Samples were analyzed for molecules involved in uptake of FAs and β-oxidation (CD36, SCARB1, SCARB2, CPT1α, PPARα, PPARγ, FABP5) in isolated NK cells **(F)**. At least five mice from two independent experiments were used. Statistically significant differences were analyzed by an unpaired t test (CPT1α, SCARB1, SCARB2) or Mann-Whitney test (CD36, PPARα, PPARγ, FABP5). Data are presented as mean values ± SEM.

CD36 is expressed by a variety of myeloid immune cells, B cells as well as NK cells (17, 30, 31) and acts to facilitate transport of long-chain FAs into cells (32). Interestingly, we show here that a subset of NK cells expresses a low level of CD36 and that CD36 expression is induced during acute infection with FV (**Figures 3A, B**). Moreover, we divided NK cells in a CD36^high^ and CD36^low^ population (**Figure 3C**) and analyzed the activation, proliferation, cytotoxicity and metabolic markers (**Figure 3D**) in CD36^high^ and CD36^low^ NK cells from FV-infected mice. CD36^high^ NK cells strongly increased their proliferation (KI-67), which peaked at 7 dpi (**Supplementary Figure 5A**) and activation (CD69). Also, cytotoxicity markers, such as TRAIL and GzmB, were highly enriched in CD36^high^ NK cells. CD36^high^ NK cells produced more IFN-γ and increased the expression of several metabolic markers such as the metabolic master regulator cMyc, the transferrin receptor CD71, CD98, which is important for amino acid transport (**Figure 3D**). Interestingly, CD36^high^ NK cells had a higher puromycin expression compared to CD36^low^ NK cells from acute FV infection (**Supplementary Figure 5B**). To confirm that NK cells increase the uptake of FAs in line with increased expression of CD36, we analyzed the ability of NK cells to take up FAs. Therefore, we incubated NK cells with Bodipy FL C16 and analyzed the FAs uptake over time in FV infection. We found that the maximum uptake of FAs by NK cells was at 7 dpi, which decreased until 28 dpi (**Figure 3E**). Moreover, the population of activated NK cells increased the uptake of FAs (**Supplementary Figures 3A, B**). Interestingly, there was a positive correlation between the FA uptake and activation of NK cells (**Supplementary Figure 3C**). Also, CD36^+^ NK cells have an increased incorporation of a fluorescent tagged C16 FA, Bodipy FL C16, during acute retrovirus infection compared to NK cells from naïve mice (**Supplementary Figure 3D**). Next, we analyzed the mRNA isolated from NK cells of uninfected and FV-infected animals for molecules involved in FA uptake and oxidation (CD36, CPT1α, PPARα, PPARγ, FABP5, SCARB1, SCARB2) (**Figure 3F**). Because the enrichment of NK cells was approximately 77%, we analyzed the other cell subsets that were present in the isolated fraction. We found almost similar percentages of macrophages, granulocytes and dendritic cells (DCs) in cells isolated from naïve or FV-infected animals (**Supplementary Figure 4**). We found a significant increase in the expression of FA transporter CD36 as well as 20-fold augmented CPT-1 (target for etomoxir) mRNA level in splenic NK cells isolated from acute FV-infected mice compared to NK cells from naïve mice. SCARB genes encode e.g. for scavenger receptor class B type 1 (SCARB1) or type 2 (SCARB2) and belong to the CD36-like superfamily. Proteins encoded by SCARBs are essential for cholesterol homeostasis and binding of high-density lipoprotein cholesterol. There was a 1.5-(SCARB1) and 1.4-fold (SCARB2) increase in the mRNA levels in NK cells of FV-infected mice. The transcription factor PPARα is regulated by free fatty acids and promotes uptake, use, and catabolism of FA by upregulation of genes involved in FA transport, fatty acid binding and activation, and β-oxidation (33). We found a significant increase in PPARα expression in NK cells from acute FV infection. PPARγ is a ligand-inducible transcription factor and regulates the expression of genes that are involved in lipid transport and metabolism such as CD36 (34). During acute FV infection, NK cells PPARγ mRNA level was significantly increased compared to naïve NK cells. Next, we analyzed the expression of small intracellular lipid chaperone Fatty acid-binding protein 5 (FABP5), which is important for FA uptake and transportation. In line with the above-mentioned genes, we found an upregulation of FABP5 in NK cells after FV infection. We hypothesized that the increased uptake of FAs takes place in organs in which FV highly replicates. Thus, we analyzed the uptake of FAs in the spleen (high virus replication) and blood (low virus replication) (**Supplementary Figure 3E**). Indeed, we found a decreased uptake of FAs in the blood. These data collectively show that NK cells increase molecules involved in FA uptake and catabolism during acute retrovirus infection.

### 3.4 Similar levels of neutral lipids in NK cells and FAs in plasma of retrovirus-infected mice compared to naïve animals

**Figure 4:**
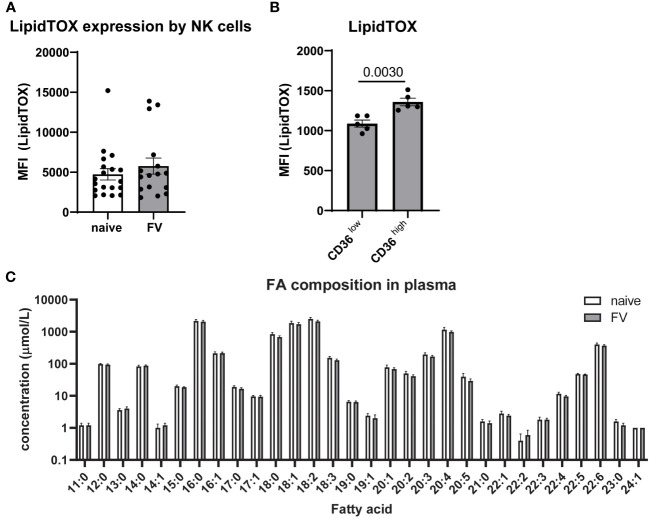
Neutral lipids in NK cells and free FAs in plasma of naïve and FV-infected mice. C57BL/6 mice were infected with FV or used as uninfected controls. NK cells of naïve or FV-infected animals were analyzed for levels of LipidTOX **(A)**. At least 16 mice from six independent experiments were used. LipidTOX staining level was analyzed in CD36^high^ and CD36^low^ NK cells **(B)**. Five animals were analyzed in two different experiments and analyzed by an unpaired t test. Plasma of five naïve and five FV-infected mice were analyzed for free and esterified FA **(C)**. Data are presented as mean values ± SEM.

Neutral lipids are important for energy storage and energy production in cells (35). We analyzed the staining of LipidTOX, which is a stain for neutral lipid droplets (**Figure 4A**). We found similar levels of LipidTOX in NK cells from naïve and FV-infected mice. Next, we were interested whether NK cells that take up FAs through CD36 accumulate FAs. Thus, we analyzed LipidTOX in CD36^high^ and CD36^low^ NK cells and found a significant increase in LipidTOX in CD36^high^ NK cells (**Figure 4B**). When we inhibited the uptake of FAs through CD36 by incubating the cells with sulfo-N-succinimidyl oleate (SSO), we found a significant decrease of LipidTOX staining level (**Supplementary Figure 6A**). Our hypothesis is that CD36^high^ NK cells increase uptake of FAs and so we wanted to exclude any differences in the overall availability of FAs in naïve mice versus FV-infected mice. For this reason, we analyzed the free and esterified FAs in the plasma of the blood. No significant differences were found in the composition of free and esterified FAs from naïve or retrovirus-infected mice (**Figure 4C**). Linoleic acid (18:2) followed by palmitic acid (16:0) and oleic acid (18:1) were present in high concentrations in the plasma of mice. Taken together, the data argues that during FV infection NK cells have an increased capacity to take up and use the FAs available in the serum.

### 3.5 Decreased killing activity of NK cells after blocking the β-oxidation

**Figure 5:**
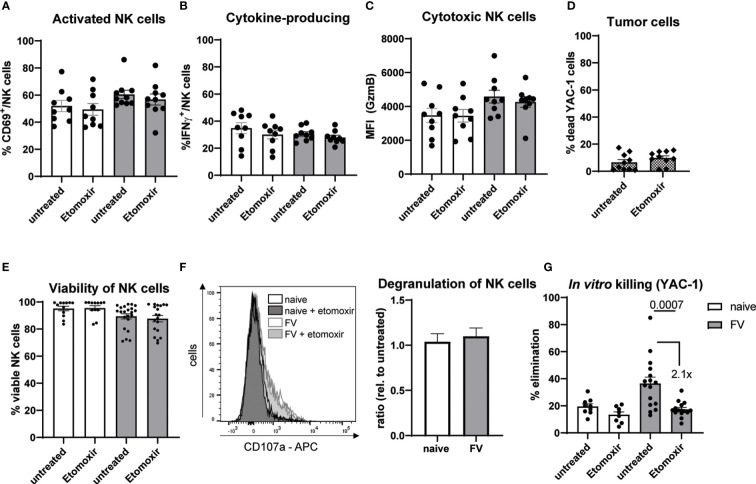
Activation and cytotoxic activity of NK cells upon acute retrovirus infection. C57BL/6 mice were infected with FV or used as uninfected controls. Splenocytes were harvested and incubated with low dose IL-15 (12.5 ng/ml) for 48 hours. Etomoxir (5 µM) was added, if indicated. NK cells were activated for 24 hours with IL-2 (20 ng/ml) and IL-12 (10 ng/ml), which were added after 24 hours of IL-15 cultivation. Total incubation time was 48 hours. NK cell activation of FV-infected animals was analyzed in **(A)** by CD69. Cells were treated with BFA (2 µg/ml) and monensin (1x) three hours in advance of IFN-γ staining **(B)**. GzmB was stained and analyzed by flow cytometer **(C)**. At least nine mice from three independent experiments were used. YAC-1 cells were stained with CFSE and incubated for 4 hours with or without etomoxir **(D)**. At least ten values from ten independent experiments were used. NK cells were isolated using magnetic beads and cultured for 4 hours with etomoxir. Viability of NK cells is shown in **(E)** At least 12 mice from seven independent experiments were used. Isolated NK cells were coincubated with CFSE-stained YAC-1 cells for 4 hours. If indicated, etomoxir was added to coculture. A representative histogram of CD107a expression of NK cells after 4 h coincubation and the relative degranulation of etomoxir treated and untreated conditions are shown in **(F)** Pooled data from six independent experiments were used. Killing capacity of NK cells was detected by flow cytometry **(G)**. At least eight animals from three independent experiments were used and analyzed by an Ordinary one-way ANOVA. Data are presented as mean values ± SEM.

We have found a decreased protein translation, assuming a decreased generation of ATP, after blocking the β-oxidation (**Figures 2E, F**) and augmented mRNA levels of molecules involved in β-oxidation (**Figure 3F**). Since long-chain FAs cannot freely enter the mitochondrial matrix, they depend on CPT-1 for transport. Hence, we inhibited the β-oxidation by using low concentration of etomoxir (5 µM), which stops the transport of FA into the mitochondria. Etomoxir-treated NK cells showed similar levels of activation, cytokine-production as well as GzmB expression compared to untreated NK cells (**Figures 5A–C**). Nevertheless, we hypothesized that etomoxir has a detrimental effect on the killing capacity of NK cells. Thus, we analyzed the killing capacity of NK cells treated with etomoxir *in vitro* (**Figures 5D–G**). We first analyzed the influence of etomoxir on target cells (YAC-1) and found no effects on the viability of tumor target cells (**Figure 5D**). Next, we analyzed the influence of etomoxir on the viability of NK cells. We did not detect a decrease in viability after etomoxir treatment compared to untreated NK cells (**Figure 5E**). Next, we analyzed the degranulation of NK cells and did not observe any effects of etomoxir treatment on NK cell degranulation (**Figure 5F**). Interestingly, CD36^high^ NK cells exhibited a higher degranulation compared to CD36^low^ NK cells upon acute infection (**Supplementary Figure 5C**). Nevertheless, we found a decreased target cell killing by NK cells treated with etomoxir. Here, NK cells from infected mice showed a more than 2-fold reduction in killing capacity. Besides treatment with etomoxir, the blockade of CD36 by SSO resulted in a significant reduction of NK cell killing (**Supplementary Figure 6B**). Collectively, these data demonstrate the importance of β-oxidation for NK cells killing abilities.

### 3.6 Inhibition of FA processing does not influence the conjugate formation but the migration of NK cells during acute retrovirus infection

**Figure 6:**
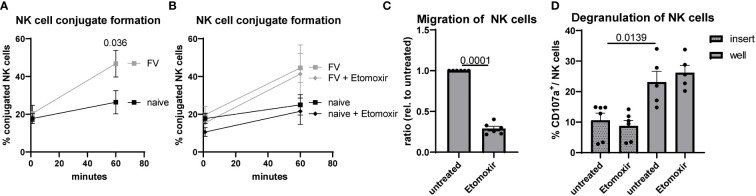
Conjugate formation and migration of NK cells after acute FV infection. C57BL/6 mice were infected with FV or used as naïve controls. Splenocytes were harvested and NK cells were isolated using magnetic beads. NK cells were stained with Tag-it Violet and were coincubated with CFSE-labeled YAC-1 cells in an E:T ratio of 1:2. Tag-it Violet^+^ CFSE^+^ conjugates were detected by flow cytometry **(A)**. If indicated, etomoxir (40 µM) was added to coculture **(B)**. At least six mice from four independent experiments were used and analyzed by an unpaired t test. In **(C)**, NK cells were seeded in the upper part of transwell whereas YAC-1 cells were seeded at the bottom. If indicated, etomoxir (40 µM) was added to coculture. After four hours, migration of NK cells into the bottom well was detected by flow cytometry. At least six animals from three independent experiments were used and analyzed by an unpaired t test. In **(D)**, migrated NK cells from the bottom well of FV-infected animals were analyzed by flow cytometry for CD107a. At least five animals from three independent experiments were used and analyzed by an unpaired t test. Data are presented as mean values ± SEM.

The process of killing a target cell is a multi-step process (36). First, NK cells have to migrate and recognize target cells. Second, after adhesion an immunological synapse has to be formed. Third, target cell can be killed by release of cytotoxic substances into this gap. Because we have seen defects in target cell killing after etomoxir treatment of NK cells (**Figure 5G**) but no differences in NK cells GzmB expression (**Figure 5C**), we asked if we find alterations in conjugate formation or migration of NK cells. So, we went one step back and analyzed the conjugate formation of NK cells with target cells (YAC-1). NK cells isolated from retrovirus-infected mice exhibited a significant higher conjugate formation compared to NK cells from naïve mice (**Figure 6A**), but treatment with etomoxir did not alter the conjugate formation in both analyzed experimental groups (**Figure 6B**). Then we went one further step back and analyzed the migratory capacity of NK cells. Migration of NK cells was almost zero when incubated without attractants. Thus, we used YAC-1 cells and plated them in the compartment below. We found a significant reduction in the migration of NK cells after etomoxir treatment in NK cells isolated from FV-infected mice (**Figure 6C**). We analyzed the degranulation of NK cells in the inserts (no migration) and wells (migrated) and did not detect differences between etomoxir-treated or untreated groups (**Figure 6D**). Although we found similar percentages of CD36^high^ and CD36^low^ subsets in the insert and well (**Supplementary Figure 5D**), we detected an increased degranulation of migrated CD36^high^ NK cells (**Supplementary Figures 5E, F**). Interestingly, the migration of CD36^high^ NK cells was strongly decreased after etomoxir treatment (**Supplementary Figure 5G**). Together, this data show that the conjugate formation is not impaired but the migration is diminished after inhibiting the β-oxidation in NK cells from FV infection.

## 4 Discussion

Viruses cause major health issues as it was recently seen for the “new” virus severe acute respiratory syndrome coronavirus type 2 (SARS-CoV-2). However, long-known viruses such as HIV, which was identified 1983, still challenge the human population worldwide. Viruses hijack and manipulate cellular pathways and in case of HIV infection, persist life-long in cells that are not affected by antiretroviral treatment. CD4^+^ T cells harbor HIV and interestingly, an augmented cellular metabolism (glycolysis, OXPHOS) was associated with increased risk of HIV infection, showing the importance of cellular metabolism for immune cells as well as viruses (37). NK cells recognize virus-infected cells and are important to fight viral infections such as infections with HIV as well as FV (20). Here we demonstrate for the first time that NK cells require FAs for their killing function as they enable NK cell migration during acute retrovirus infection. We have used the well-characterized FV mouse model to analyze the uptake of FAs during course of infection and show the ability of NK cells to use FAs to fuel their metabolic demands. NK cells circulate through the body and enter different organs and tissues. Every microenvironment has different levels of fuels and oxygen. We have shown that NK cells are highly activated and cytotoxic after acute retrovirus infection and increase the uptake of nutrients (25). We have metabolically profiled NK cells with the so-called method single cell energetic metabolism by profiling translation inhibition (SCENITH) (28). Analog to our previous study, ATP content of NK cells from FV-infected mice was increased compared to NK cells from uninfected mice when analyzed with SCENITH (25). Moreover, the capacity to use FAs or amino acids as fuel was substantially higher in NK cells from FV infection. Thus, the ability to utilize multiple fuels such as glucose or fatty acids is beneficial and required for their function in virus infections. In 2015 Keppel et al. demonstrated *in vitro* that cytokine-activated as well as receptor-activated NK cells use glucose as major fuel for OXPHOS and inhibition of β-oxidation by etomoxir, which is the rate-limiting enzyme for mitochondrial import of long-chain FAs, had no influence on NK cells ATP synthesis (16). Here we show in a mouse model that NK cells are able to increase their glycolysis but at the same time have the flexibility to change their fuel to utilize FAs as well as amino acids as they need the plasticity to adapt to different tissues with different nutrient and oxygen availability.

In 2018, obesity was associated with decreased NK cell cytotoxicity (17). It was shown that the memory formation of NK cells is impaired in people with obesity (38). Furthermore, Kobayashi et al. demonstrated that increased lipid metabolism resulted in impaired cytolytic NK cell responses in aggressive B cell lymphoma (18). However, we show here that FAs are important for the NK cell activity in acute virus infection. A major difference to the above-mentioned studies is the duration of FA stimulation. We have analyzed NK cells in short-term, acute FV infection in healthy mice in comparison to the studies with constant FA stimulation as it is seen in obesity or FA-rich lymphoma.

One important regulator of lipid metabolism is mTOR. Despite regulating the FA and lipid metabolism, mTOR signaling also coordinates amino acid, glucose and nucleotide metabolic programs. NK cells require mTOR for robust production of IFN-γ (39). During the course of FV infection, NK cells start to increase their mTOR expression, as we detected by the phosphorylated downstream signaling target S6. During acute FV infection, NK cells increase their cytokine production and release, indicating an important function of mTOR during acute virus infections (25). Beside cytokine production, NK cells have to migrate to target cells in order to kill target cells, recognize targets and form an immunological synapse, in which they can release their cytotoxic molecules (36). Remarkably, NK cells that have defects in FA oxidation had an impaired migratory capacity, but no alterations in conjugate formations.

β-oxidation is crucial for the generation of memory CD8^+^ T cells (12), M2 macrophages (40) and Tregs (13). However, the early studies of FA catabolism blocked the β-oxidation by using high concentrations of etomoxir (up to 1 mM), which has been shown to have multiple off-target effects (10, 41, 42). Yao et al. found an antiproliferative effect of etomoxir used in high concentrations (up to 200 μM) for cancer cell lines independent of β-oxidation. High concentrations of etomoxir inhibited complex I of the electron transport chain (10). Low concentration of etomoxir (10 μM) impaired approximately 90% of β-oxidation in cancer cells. Moreover, 5 μM of etomoxir was shown by O’Connor et al. to specifically inhibit the oxidative metabolism in T cells (41). We treated our splenocytes with either 5 μM or 40 μM and did not observe antiproliferative effects as seen by KI-67^+^ NK cells for both concentrations (**Supplementary Figure 2**) suggesting that etomoxir treatment influences indeed the β-oxidation and not the proliferation due to off-target effects in NK cells. Interestingly, by using genetically abrogated CPT-1a expression in T cells, CPT-1a was largely dispensable for memory CD8^+^ T cells or Treg generation (42). However, CPT-1a-deficient T cells are still able to oxidize other FAs such as short-chain FAs, which may serve as additional fuels and may compensate for the loss of long-chain FAs. Beside FAs, amino acids such as glutamine can be used to fuel T cells intracellular FA production (41). We have shown that NK cells have an increased fuel flexibility and are able to switch to FA oxidation as well as amino acid oxidation upon FV infection. By blocking the β-oxidation, we showed that NK cells require FAs for their migratory capacity upon acute FV infection. Additionally, we have demonstrated that activated NK cells enhanced their uptake of long-chain FAs, expression of the rate-limiting FA uptake receptor CD36 as well as increased their levels of small intracellular lipid chaperone FABP5 during acute FV infection contradictory to memory CD8^+^ T cells as well as T helper 17 cells that acquire substantially less long-chain FAs from their environment (14, 15). Memory CD8^+^ T cells use extracellular glucose for *de novo* FA synthesis to support β-oxidation rather than uptake of extracellular FAs. Interestingly, Tregs take up exogeneous FAs (14). Similar to memory CD8^+^ T cells, we found an increased capacity to engage in glycolysis in NK cells from acute FV infection, which is important in low oxygen environment. However, we found an increased uptake of FAs by NK cells in organs of high FV replication such as the spleen, but not in NK cells from blood. Michelet et al. showed that accumulation of lipids in NK cells e.g. in obese individuals resulted in impaired NK cells GzmB expression (17). Interestingly, we detected an accumulation of neutral lipids in NK cells that actively take up FAs in mice acutely infected with FV. These NK cells express high levels of CD36 and were metabolically more active and cytotoxic. Hence, this likely reflects that NK cells from FV-infected mice can store some lipids to a certain extent without losing their cytotoxic functions. Interestingly, we did not detect alterations in the composition of free FAs in plasma indicating that NK cells take up FAs as they require for their metabolic needs to perform target cell killing during retrovirus infection.

Having characterized the substantive importance of FA metabolism on NK cell cytotoxicity, it is clear that FAs are far more than simple energy storage. We have demonstrated that the FA uptake and oxidation are essential for the activity of NK cells during acute retrovirus infection. New therapeutic approaches targeting the lipid metabolism, either by manipulating the FA immunonutrition to promote an improved immune response or by modulating metabolic parameters involved in lipid metabolism, might be considered in the future.

## Data availability statement

The raw data supporting the conclusions of this article will be made available by the authors, without undue reservation.

## Ethics statement

The animal study was approved by State Agency for Nature, Environment and Consumer Protection (LANUV). The study was conducted in accordance with the local legislation and institutional requirements.

## Author contributions

SS: Investigation, Writing – review & editing. TW: Investigation, Writing – review & editing. PG: Investigation, Writing – review & editing. SM: Formal Analysis, Investigation, Writing – review & editing. DM: Investigation, Writing – review & editing. DF: Methodology, Writing – review & editing. UD: Writing – review & editing. EL: Conceptualization, Formal Analysis, Funding acquisition, Investigation, Project administration, Resources, Supervision, Validation, Visualization, Writing – original draft, Writing – review & editing.

## Supplementary material

Supplementary Figure 1 Etomoxir-mediated reduction of energy levels in NK cells upon acute FV infection. C57BL/6 mice were infected with FV or used as naïve controls. Spleens were harvested and homogenized. For SCENITH analysis, splenocytes were treated with etomoxir (5 μM, 40 μM), 2-DG (100 mM) or oligomycin (1 μM). NK cells were incubated and stained for puromycin. At least five mice from two independent experiments were used. Statistically significant differences were analyzed by an Ordinary one-way ANOVA. In B, NK cells were isolated and 200,000 NK cells were attached with CellTAK (Corning). Oligomycin (2 µM), Carbonyl cyanide-p-trifluoro¬methoxyphenyl¬hydrazone (FCCP, 1 μM final), etomoxir (5 μM, 40 μM) and rotenone/antimycin A (0.1 µM final; 4 µM final) were injected. Samples were run at Seahorse analyzer XFp (Agilent). At least five mice from five independent experiments were used. Statistically significant differences were analyzed by a Mann-Whitney test. Data are presented as mean values ± SEM.

Supplementary Figure 2 Proliferation of NK cells after treatment with etomoxir in FV-infected mice. C57BL/6 mice were infected with FV for 7 days. Splenocytes were harvested and incubated with low dose IL-15 (12.5 ng/ml) for 48 hours. Etomoxir (5 μM or 40 μM) were added for the last 24 hours, if indicated. NK cells were activated for 24 hours with IL-2 (20 ng/ml) and IL-12 (10 ng/ml). Proliferation of NK cells was measured with KI-67. Data are presented as mean values ± SEM. At least five mice from two independent experiments were used and presented as mean values ± SEM.

Supplementary Figure 3 FA uptake by NK cells upon acute FV infection. C57BL/6 mice were infected with FV or used as naïve controls. Splenocytes were harvested and incubated with BODIPY FL C16. NK cells were stained for CD69 and were measured using flow cytometry. A representative histogram of BODIPY FL C16 and CD69 expressing NK cells is shown in A. CD69^+^BODIPY FL C16^+^ NK cells were shown in B as bar graph. Data are presented as mean values ± SEM. At least ten mice from six independent experiments were used and analyzed by a Mann-Whitney test. In C, correlation of BODIPY FL C16 and CD69 in NK cells is shown. Percentage of CD36^+^ BODIPY FL C16^+^ NK cells is shown in D. At least 12 mice from four independent experiments were used and analyzes by an unpaired t test. (E) NK cells uptake of FA in spleens (high virus replication) and blood (low viral replication) of 7 dpi-infected mice. At least four mice from two independent experiments were used and analyzed by an unpaired t test. Data are presented as mean values ± SEM.

Supplementary Figure 4 Cell subsets after NK cell isolation. C57BL/6 mice were infected with FV or used as naïve mice. Splenic NK cells were isolated with magnetic beads (MojoSort, BioLegend) according to the manufacturer’s instructions. Isolated cells were stained for NK cells (CD3-NK1.1^+^), B cells (CD3^-^NK1.1^-^ CD19^+^), DCs (CD3^-^NK1.1^-^CD11c^+^), granulocytes (CD3^-^NK1.1^-^Gr1^+^), macrophages (CD3^-^NK1.1^-^CD11b^+^F4/80^+^) and T cells (CD3^+^NK1.1^-^). At least four animals from two independent experiments were used. Data are presented as mean values ± SEM.

Supplementary Figure 5 Characterization of CD36^high^ and CD36^low^ NK cells upon FV infection. C57BL/6 mice were infected with FV and spleens were harvested at 3, 7 and 21 dpi. Splenic NK cells were stained and analyzed for KI-67 (A). At least two animals (3 dpi) were used and 7 dpi (5 mice) was analyzed by an unpaired t test. In B, SCENITH assay was performed and the expression of puromycin was analyzed in CD36^high^ and CD36^low^ NK cells. At least 18 animals were used from four independent experiments and analyzed by an unpaired t test. The degranulation of NK cells was analyzed at the end of *in vitro* kill assay by detection of CD107a (C). At least 15 mice from five independent experiments were used and analysed by an unpaired t test. Splenic NK cells were isolated with magnetic beads and plated into the insert of a transwell. YAC-1 cells were seeded as attractant in the well below. NK cells were coincubated with YAC-1 cells for 4 hours. After 4 hours, NK cells (D) and the percentage of CD107a^+^ NK cells was analyzed in the insert and well (E, F). The migration of untreated and etomoxir treated CD36^high^ NK cells was calculated in G. At least six animals from three independent experiments were used and analyzed by an unpaired t test. Data are presented as mean values ± SEM.

Supplementary Figure 6 Influence of CD36 blockade on NK cells after acute FV infection. C57BL/6 mice were infected with FV. Splenocytes were incubated for 30 min with SSO (200 µM) and stained for NK cell markers and LipidTOX (A). At least six animals from two independent experiments were used and analyzed by a Mann-Whitney test. NK cells were isolated and YAC-1 cells were stained with CFSE. To block CD36, SSO (200 µM) was added to coculture. After 4 hours, killing was detected at flow cytometer (B). At least six mice (FV infection) from two independent experiments were used. Statistically significant differences were analyzed by an unpaired t test. Data are presented as mean values ± SEM.

## References

[1] Orange JS. Formation and function of the lytic NK-cell immunological synapse. Nat Rev Immunol (2008) 8:713–25. doi: 10.1038/nri2381 PMC2772177 19172692

[2] Vivier E Tomasello E Baratin M Walzer T Ugolini S. Functions of natural killer cells. Nat Immunol (2008) 9:503–10. doi: 10.1038/ni1582 18425107

[3] Biron CA Nguyen KB Pien GC Cousens LP Salazar-Mather TP. Natural killer cells in antiviral defense: function and regulation by innate cytokines. Annu Rev Immunol (1999) 17:189–220. doi: 10.1146/annurev.immunol.17.1.189 10358757

[4] O'Brien KL Finlay DK. Immunometabolism and natural killer cell responses. Nat Rev Immunol (2019) 19:282–90. doi: 10.1038/s41577-019-0139-2 30808985

[5] Donnelly RP Finlay DK. Glucose, glycolysis and lymphocyte responses. Mol Immunol (2015) 68:513–9. doi: 10.1016/j.molimm.2015.07.034 26260211

[6] Assmann N O'brien KL Donnelly RP Dyck L Zaiatz-Bittencourt V Loftus RM. Srebp-controlled glucose metabolism is essential for NK cell functional responses. Nat Immunol (2017) 18:1197–206. doi: 10.1038/ni.3838 28920951

[7] Arnold PK Jackson BT Paras KI Brunner JS Hart ML Newsom OJ. A non-canonical tricarboxylic acid cycle underlies cellular identity. Nature (2022) 603:477–81. doi: 10.1038/s41586-022-04475-w PMC8934290 35264789

[8] Caro-Maldonado A Gerriets VA Rathmell JC. Matched and mismatched metabolic fuels in lymphocyte function. Semin Immunol (2012) 24:405–13. doi: 10.1016/j.smim.2012.12.002 PMC3582857 23290889

[9] Schreurs M Kuipers F van der Leij FR. Regulatory enzymes of mitochondrial beta-oxidation as targets for treatment of the metabolic syndrome. Obes Rev (2010) 11:380–8. doi: 10.1111/j.1467-789X.2009.00642.x 19694967

[10] Yao CH Liu GY Wang R Moon SH Gross RW Patti GJ. Identifying off-target effects of etomoxir reveals that carnitine palmitoyltransferase I is essential for cancer cell proliferation independent of beta-oxidation. PloS Biol (2018) 16:e2003782. doi: 10.1371/journal.pbio.2003782 29596410 PMC5892939

[11] Lochner M Berod L Sparwasser T. Fatty acid metabolism in the regulation of T cell function. Trends Immunol (2015) 36:81–91. doi: 10.1016/j.it.2014.12.005 25592731

[12] Pearce EL Walsh MC Cejas PJ Harms GM Shen H Wang LS. Enhancing CD8 T-cell memory by modulating fatty acid metabolism. Nature (2009) 460:103–7. doi: 10.1038/nature08097 PMC2803086 19494812

[13] Michalek RD Gerriets VA Jacobs SR Macintyre AN Maciver NJ Mason EF. Cutting edge: distinct glycolytic and lipid oxidative metabolic programs are essential for effector and regulatory CD4+ T cell subsets. J Immunol (2011) 186:3299–303. doi: 10.4049/jimmunol.1003613 PMC3198034 21317389

[14] Berod L Friedrich C Nandan A Freitag J Hagemann S Harmrolfs K. *De novo* fatty acid synthesis controls the fate between regulatory T and T helper 17 cells. Nat Med (2014) 20:1327–33. doi: 10.1038/nm.3704 25282359

[15] O'Sullivan D van der Windt GJ Huang SC Curtis JD Chang CH Buck MD. Memory CD8(+) T cells use cell-intrinsic lipolysis to support the metabolic programming necessary for development. Immunity (2014) 41:75–88. doi: 10.1016/j.immuni.2014.06.005 25001241 PMC4120664

[16] Keppel MP Saucier N Mah AY Vogel TP Cooper MA. Activation-specific metabolic requirements for NK Cell IFN-gamma production. J Immunol (2015) 194:1954–62. doi: 10.4049/jimmunol.1402099 PMC4323953 25595780

[17] Michelet X Dyck L Hogan A Loftus RM Duquette D Wei K. Metabolic reprogramming of natural killer cells in obesity limits antitumor responses. Nat Immunol (2018) 19:1330–40. doi: 10.1038/s41590-018-0251-7 30420624

[18] Kobayashi T Lam PY Jiang H Bednarska K Gloury R Murigneux V. Increased lipid metabolism impairs NK cell function and mediates adaptation to the lymphoma environment. Blood (2020) 136:3004–17. doi: 10.1182/blood.2020005602 32818230

[19] Chesebro B Miyazawa M Britt WJ. Host genetic control of spontaneous and induced immunity to Friend murine retrovirus infection. Annu Rev Immunol (1990) 8:477–99. doi: 10.1146/annurev.iy.08.040190.002401 2188671

[20] Littwitz-Salomon E Dittmer U Sutter K. Insufficient natural killer cell responses against retroviruses: how to improve NK cell killing of retrovirus-infected cells. Retrovirology (2016) 13:77. doi: 10.1186/s12977-016-0311-8 27821119 PMC5100108

[21] Littwitz E Francois S Dittmer U Gibbert K. Distinct roles of NK cells in viral immunity during different phases of acute Friend retrovirus infection. Retrovirology (2013) 10:127. doi: 10.1186/1742-4690-10-127 24182203 PMC3826539

[22] Mavilio D Lombardo G Benjamin J Kim D Follman D Marcenaro E. Characterization of CD56-/CD16+ natural killer (NK) cells: a highly dysfunctional NK subset expanded in HIV-infected viremic individuals. Proc Natl Acad Sci U.S.A. (2005) 102:2886–91. doi: 10.1073/pnas.0409872102 PMC549494 15699323

[23] Reeves RK Li H Jost S Blass E Schafer JL Varner V. Antigen-specific NK cell memory in rhesus macaques. Nat Immunol (2015) 16:927–32. doi: 10.1038/ni.3227 PMC4545390 26193080

[24] Littwitz-Salomon E Nguyen T Schimmer S Dittmer U. Friend retrovirus infection induces the development of memory-like natural killer cells. Retrovirology (2018) 15:68. doi: 10.1186/s12977-018-0450-1 30292240 PMC6174066

[25] Littwitz-Salomon E Moreira D Frost JN Choi C Liou KT Ahern DK. Metabolic requirements of NK cells during the acute response against retroviral infection. Nat Commun (2021) 12, 5376. doi: 10.1038/s41467-021-25715-z 34508086 PMC8433386

[26] Matyash V Liebisch G Kurzchalia TV Shevchenko A Schwudke D. Lipid extraction by methyl-tert-butyl ether for high-throughput lipidomics. J Lipid Res (2008) 49:1137–46. doi: 10.1194/jlr.D700041-JLR200 PMC2311442 18281723

[27] Görs PE Wittenhofer P Ayala-Cabrera JF Meckelmann SW. Potential of atmospheric pressure ionization sources for the analysis of free fatty acids in clinical and biological samples by gas chromatography-mass spectrometry. Anal Bioanal Chem (2022) 414:6621–34. doi: 10.1007/s00216-022-04223-z PMC9411222 35851410

[28] Arguello RJ Combes AJ Char R Gigan JP Baaziz AI Bousiquot E. SCENITH: A flow cytometry-based method to functionally profile energy metabolism with single-cell resolution. Cell Metab (2020) 32:1063–75.e7. doi: 10.1016/j.cmet.2020.11.007 33264598 PMC8407169

[29] Fritz IB Yue KT. Long-chain carnitine acyltransferase and the role of acylcarnitine derivatives in the catalytic increase of fatty acid oxidation induced by carnitine. J Lipid Res (1963) 4:279–88. doi: 10.1016/S0022-2275(20)40302-5 14168165

[30] Niavarani SR Lawson C Bakos O Boudaud M Batenchuk C Rouleau S. Lipid accumulation impairs natural killer cell cytotoxicity and tumor control in the postoperative period. BMC Cancer (2019) 19:823. doi: 10.1186/s12885-019-6045-y 31429730 PMC6701111

[31] Hu X Jia X Xu C Wei Y Wang Z Liu G. Downregulation of NK cell activities in Apolipoprotein C-III-induced hyperlipidemia resulting from lipid-induced metabolic reprogramming and crosstalk with lipid-laden dendritic cells. Metabolism (2021) 120:154800. doi: 10.1016/j.metabol.2021.154800 34051224

[32] Chen Y Zhang J Cui W Silverstein RL. CD36, a signaling receptor and fatty acid transporter that regulates immune cell metabolism and fate. J Exp Med (2022) 219:1. doi: 10.1084/jem.20211314 PMC9022290 35438721

[33] Kersten S. Integrated physiology and systems biology of PPARalpha. Mol Metab (2014) 3:354–71. doi: 10.1016/j.molmet.2014.02.002 PMC4060217 24944896

[34] Hernandez-Quiles M Broekema MF Kalkhoven E. PPARgamma in metabolism, immunity, and cancer: unified and diverse mechanisms of action. Front Endocrinol (Lausanne) (2021) 12:624112. doi: 10.3389/fendo.2021.624112 33716977 PMC7953066

[35] Hutchins PM Barkley RM Murphy RC. Separation of cellular nonpolar neutral lipids by normal-phase chromatography and analysis by electrospray ionization mass spectrometry. J Lipid Res (2008) 49:804–13. doi: 10.1194/jlr.M700521-JLR200 PMC2367097 18223242

[36] Mace EM Dongre P Hsu HT Sinha P James AM Mann SS. Cell biological steps and checkpoints in accessing NK cell cytotoxicity. Immunol Cell Biol (2014) 92:245–55. doi: 10.1038/icb.2013.96 PMC3960583 24445602

[37] Valle-Casuso JC Angin M Volant S Passaes C Monceaux V Mikhailova A. Cellular metabolism is a major determinant of HIV-1 reservoir seeding in CD4(+) T cells and offers an opportunity to tackle infection. Cell Metab (2019) 29:611–626 e5. doi: 10.1016/j.cmet.2018.11.015 30581119

[38] Kedia-Mehta N Tobin L Zaiatz-Bittencourt V Pisarska MM De Barra C Choi C. Cytokine-induced natural killer cell training is dependent on cellular metabolism and is defective in obesity. Blood Adv (2021) 5:4447–55. doi: 10.1182/bloodadvances.2021005047 PMC8579258 34607345

[39] Donnelly RP Loftus RM Keating SE Liou KT Biron CA Gardiner CM. mTORC1-dependent metabolic reprogramming is a prerequisite for NK cell effector function. J Immunol (2014) 193:4477–84. doi: 10.4049/jimmunol.1401558 PMC4201970 25261477

[40] Rosa Neto JC Calder PC Curi R Newsholme P Sethi JK Silveira LS. The immunometabolic roles of various fatty acids in macrophages and lymphocytes. Int J Mol Sci (2021) 22:3–4. doi: 10.3390/ijms22168460 PMC8395092 34445165

[41] O'Connor RS Guo L Ghassemi S Snyder NW Worth AJ Weng L. The CPT1a inhibitor, etomoxir induces severe oxidative stress at commonly used concentrations. Sci Rep (2018) 8:6289. doi: 10.1038/s41598-018-24676-6 29674640 PMC5908836

[42] Raud B Roy DG Divakaruni AS Tarasenko TN Franke R Ma EH. Etomoxir actions on regulatory and memory T cells are independent of Cpt1a-mediated fatty acid oxidation. Cell Metab (2018) 28:504–515 e7. doi: 10.1016/j.cmet.2018.06.002 30043753 PMC6747686

