# Supplementary figures and images for: Fatty acids are crucial to fuel NK cells upon acute retrovirus infection
Source: Front Immunol. 2023 Nov 29;14:1296355. doi: 10.3389/fimmu.2023.1296355 (PMC10716207; doi:10.3389/fimmu.2023.1296355)

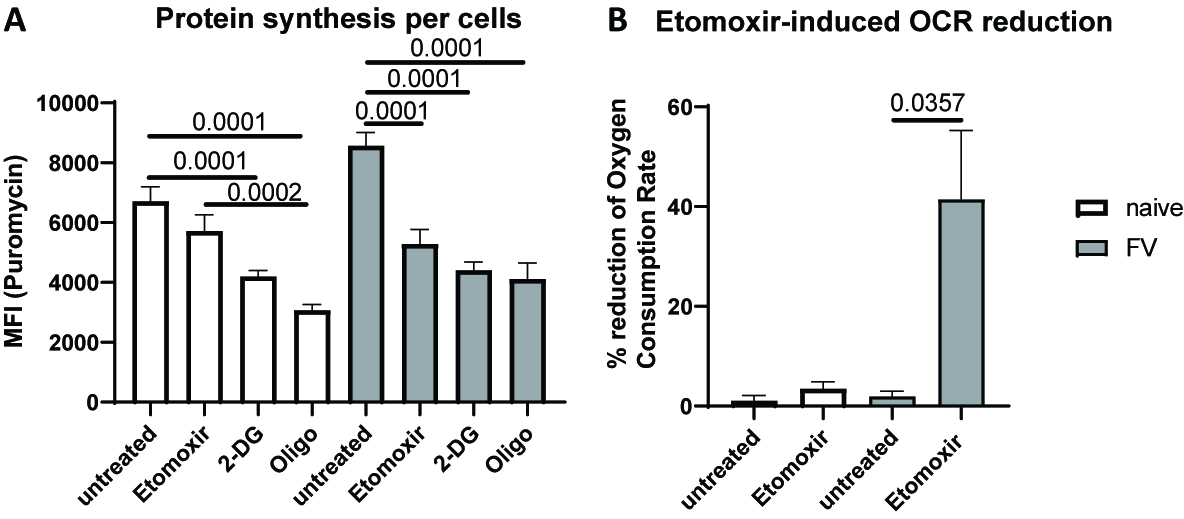

Supplement: Supplementary Figure 1 — Etomoxir-mediated reduction of energy levels in NK cells upon acute FV infection. C57BL/6 mice were infected with FV or used as naïve controls. Spleens were harvested and homogenized. For SCENITH analysis, splenocytes were treated with etomoxir (5 μM, 40 μM), 2-DG (100 mM) or oligomycin (1 μM). NK cells were incubated and stained for puromycin. At least five mice from two independent experiments were used. Statistically significant differences were analyzed by an Ordinary one-way ANOVA. In B, NK cells were isolated and 200,000 NK cells were attached with CellTAK (Corning). Oligomycin (2 µM), Carbonyl cyanide-p-trifluoro¬methoxyphenyl¬hydrazone (FCCP, 1 μM final), etomoxir (5 μM, 40 μM) and rotenone/antimycin A (0.1 µM final; 4 µM final) were injected. Samples were run at Seahorse analyzer XFp (Agilent). At least five mice from five independent experiments were used. Statistically significant differences were analyzed by a Mann-Whitney test. Data are presented as mean values ± SEM. [file Image_1.tif]

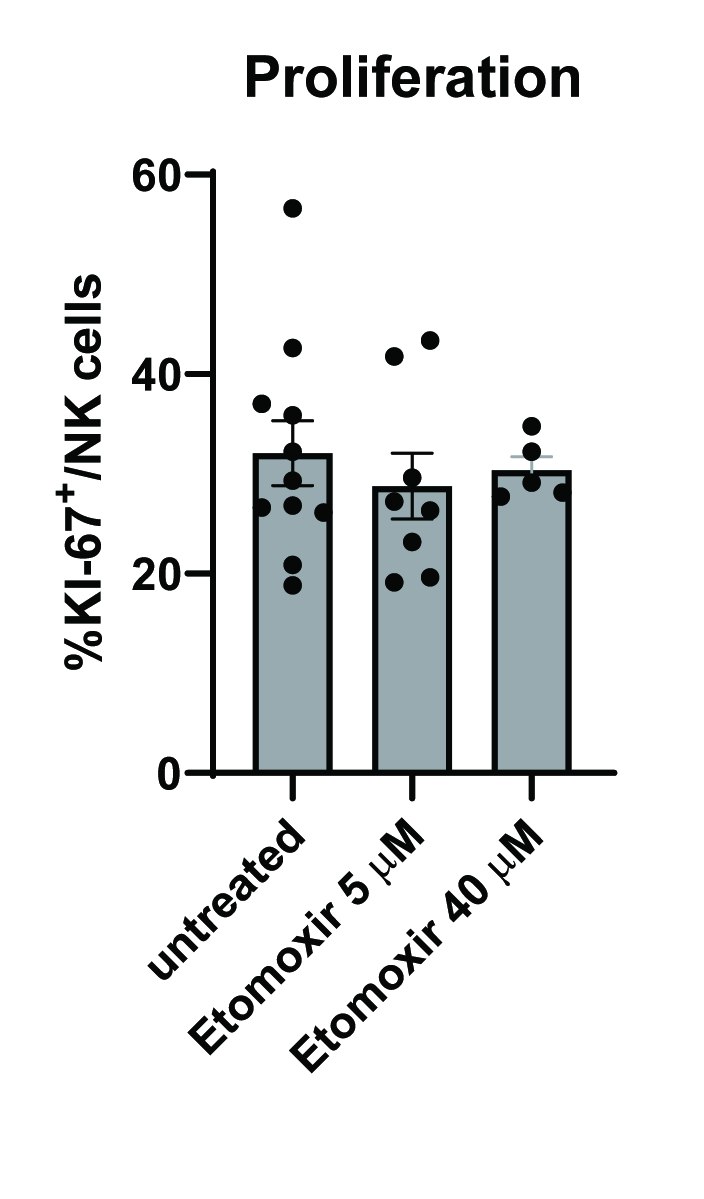

Supplement: Supplementary Figure 2 — Proliferation of NK cells after treatment with etomoxir in FV-infected mice. C57BL/6 mice were infected with FV for 7 days. Splenocytes were harvested and incubated with low dose IL-15 (12.5 ng/ml) for 48 hours. Etomoxir (5 μM or 40 μM) were added for the last 24 hours, if indicated. NK cells were activated for 24 hours with IL-2 (20 ng/ml) and IL-12 (10 ng/ml). Proliferation of NK cells was measured with KI-67. Data are presented as mean values ± SEM. At least five mice from two independent experiments were used and presented as mean values ± SEM. [file Image_2.tif]

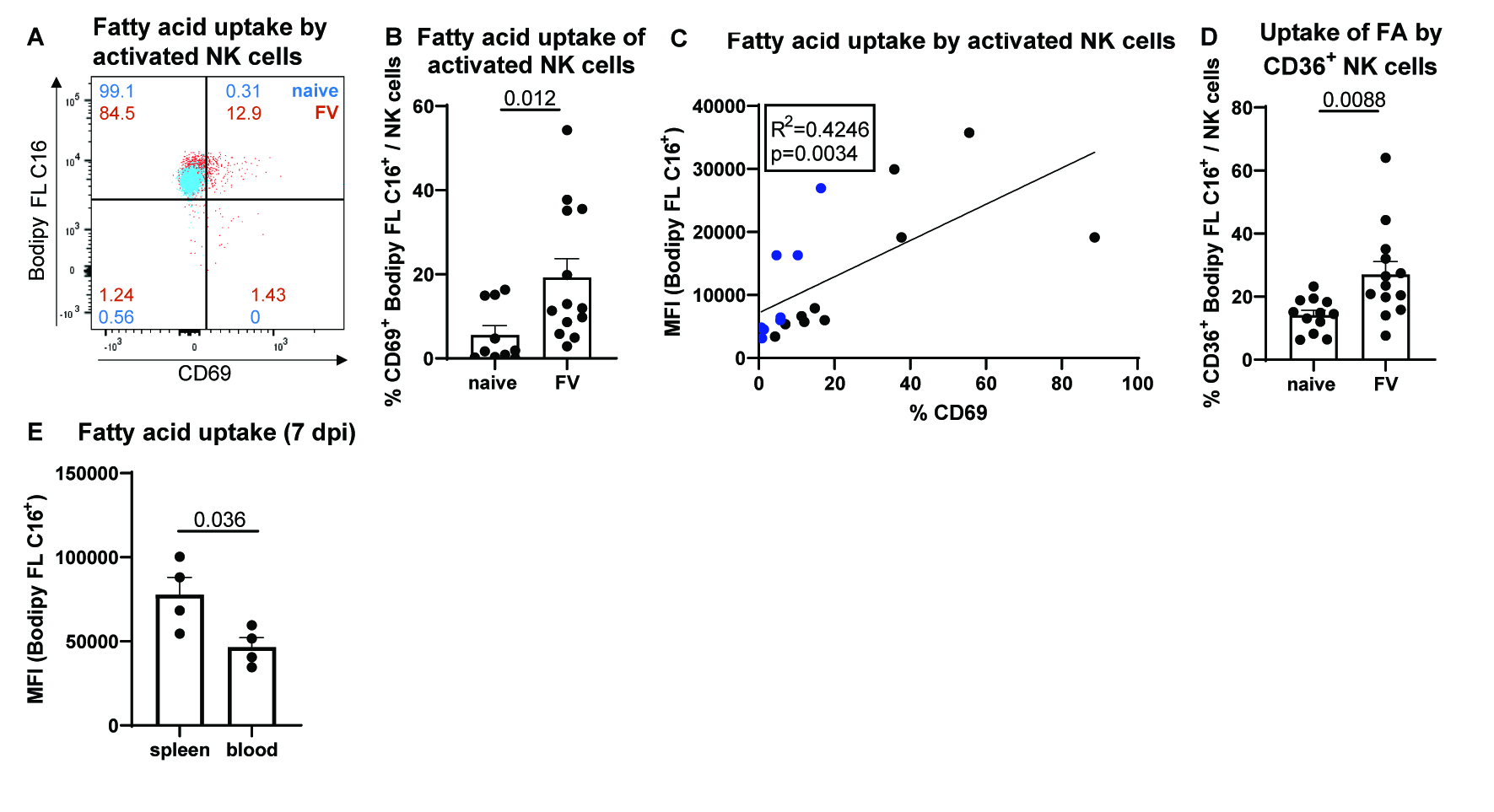

Supplement: Supplementary Figure 3 — FA uptake by NK cells upon acute FV infection. C57BL/6 mice were infected with FV or used as naïve controls. Splenocytes were harvested and incubated with BODIPY FL C16. NK cells were stained for CD69 and were measured using flow cytometry. A representative histogram of BODIPY FL C16 and CD69 expressing NK cells is shown in A. CD69+BODIPY FL C16+ NK cells were shown in B as bar graph. Data are presented as mean values ± SEM. At least ten mice from six independent experiments were used and analyzed by a Mann-Whitney test. In C, correlation of BODIPY FL C16 and CD69 in NK cells is shown. Percentage of CD36+ BODIPY FL C16+ NK cells is shown in D. At least 12 mice from four independent experiments were used and analyzes by an unpaired t test. (E) NK cells uptake of FA in spleens (high virus replication) and blood (low viral replication) of 7 dpi-infected mice. At least four mice from two independent experiments were used and analyzed by an unpaired t test. Data are presented as mean values ± SEM. [file Image_3.tif]

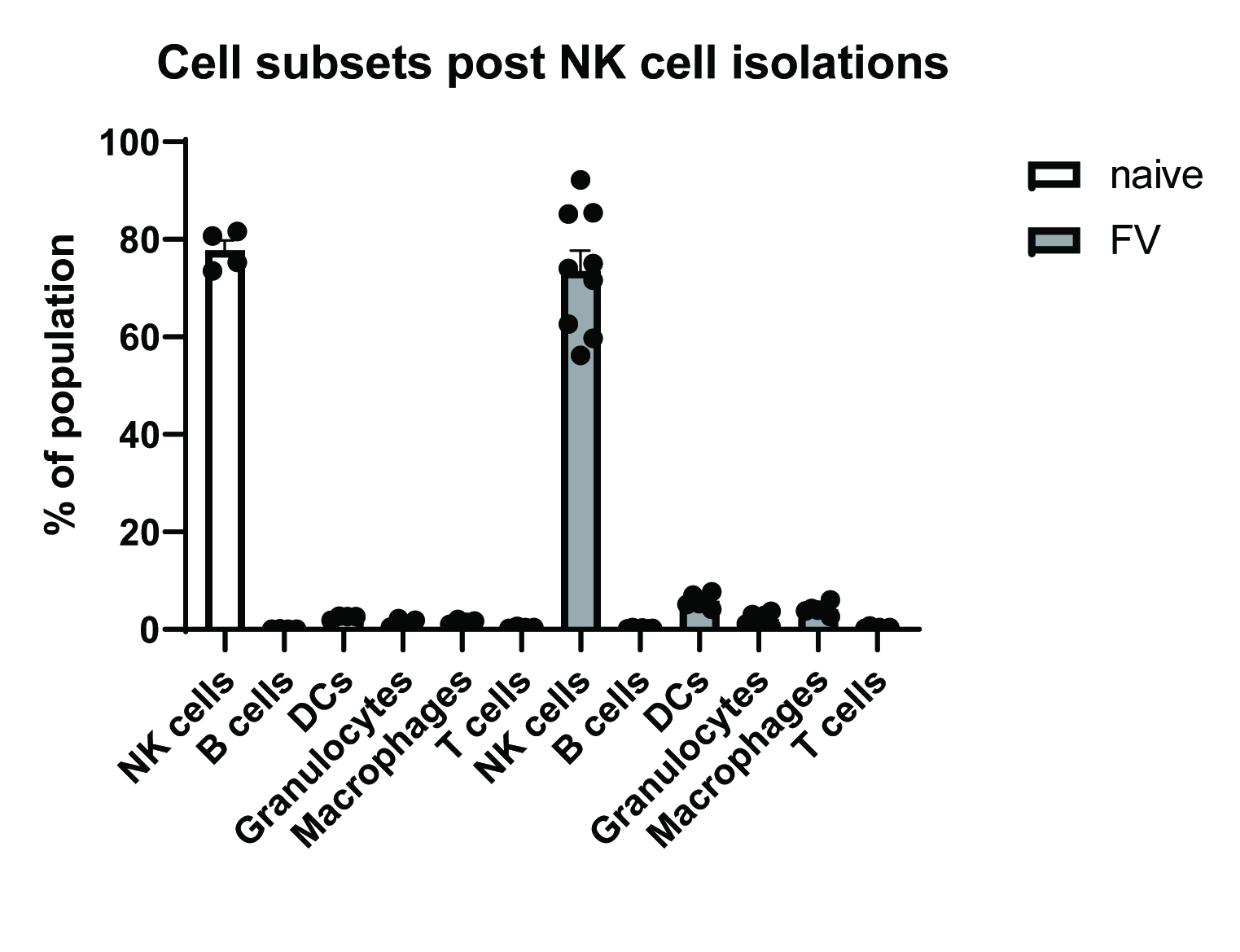

Supplement: Supplementary Figure 4 — Cell subsets after NK cell isolation. C57BL/6 mice were infected with FV or used as naïve mice. Splenic NK cells were isolated with magnetic beads (MojoSort, BioLegend) according to the manufacturer’s instructions. Isolated cells were stained for NK cells (CD3-NK1.1+), B cells (CD3-NK1.1- CD19+), DCs (CD3-NK1.1-CD11c+), granulocytes (CD3-NK1.1-Gr1+), macrophages (CD3-NK1.1-CD11b+F4/80+) and T cells (CD3+NK1.1-). At least four animals from two independent experiments were used. Data are presented as mean values ± SEM. [file Image_4.tif]

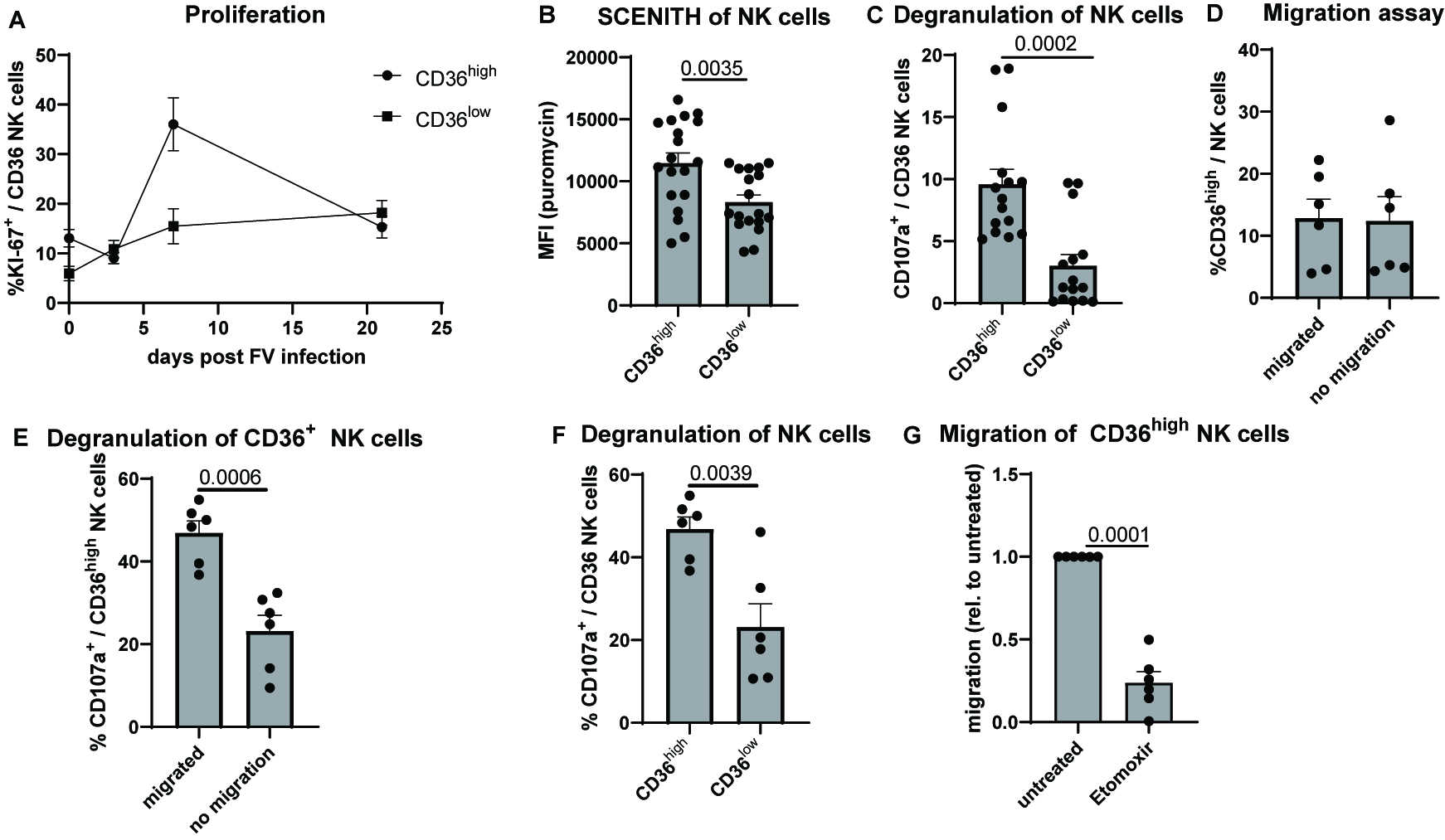

Supplement: Supplementary Figure 5 — Characterization of CD36high and CD36low NK cells upon FV infection. C57BL/6 mice were infected with FV and spleens were harvested at 3, 7 and 21 dpi. Splenic NK cells were stained and analyzed for KI-67 (A). At least two animals (3 dpi) were used and 7 dpi (5 mice) was analyzed by an unpaired t test. In B, SCENITH assay was performed and the expression of puromycin was analyzed in CD36high and CD36low NK cells. At least 18 animals were used from four independent experiments and analyzed by an unpaired t test. The degranulation of NK cells was analyzed at the end of in vitro kill assay by detection of CD107a (C). At least 15 mice from five independent experiments were used and analysed by an unpaired t test. Splenic NK cells were isolated with magnetic beads and plated into the insert of a transwell. YAC-1 cells were seeded as attractant in the well below. NK cells were coincubated with YAC-1 cells for 4 hours. After 4 hours, NK cells (D) and the percentage of CD107a+ NK cells was analyzed in the insert and well (E, F). The migration of untreated and etomoxir treated CD36high NK cells was calculated in G. At least six animals from three independent experiments were used and analyzed by an unpaired t test. Data are presented as mean values ± SEM. [file Image_5.tif]

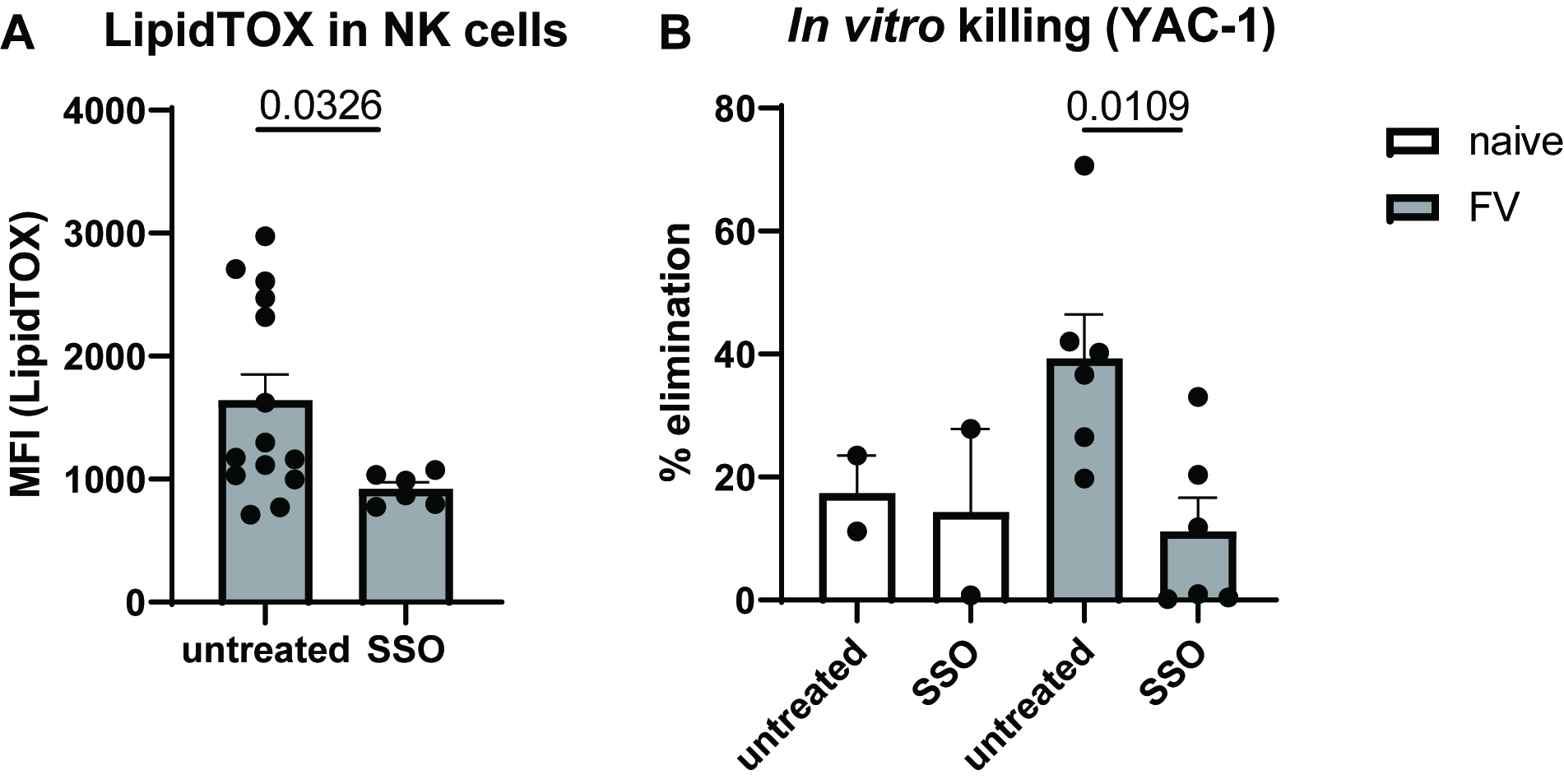

Supplement: Supplementary Figure 6 — Influence of CD36 blockade on NK cells after acute FV infection. C57BL/6 mice were infected with FV. Splenocytes were incubated for 30 min with SSO (200 µM) and stained for NK cell markers and LipidTOX (A). At least six animals from two independent experiments were used and analyzed by a Mann-Whitney test. NK cells were isolated and YAC-1 cells were stained with CFSE. To block CD36, SSO (200 µM) was added to coculture. After 4 hours, killing was detected at flow cytometer (B). At least six mice (FV infection) from two independent experiments were used. Statistically significant differences were analyzed by an unpaired t test. Data are presented as mean values ± SEM. [file Image_6.tif]
